# Perceived Social Isolation in Heart Failure

**DOI:** 10.19102/icrm.2022.130606

**Published:** 2022-06-15

**Authors:** Maria Polikandrioti

**Affiliations:** ^1^Department of Nursing, University of West Attica, Athens, Greece

**Keywords:** Heart failure, fatigue, perceived social isolation

## Abstract

Patients with heart failure (HF) experience social isolation associated with an increased risk of morbidity, mortality, and elevated health care expenditures. The aim of this study was to evaluate the factors associated with perceived social isolation and to assess the impact of fatigue on social isolation. A total of 100 HF outpatients were enrolled by convenience sampling. Data were collected by completion of the Greek version of the Modified Fatigue Impact Scale (MFIS-Greek), which also included patients’ characteristics and their self-report about social isolation. Of the 100 participants (68% men; mean age, 68.6 ± 7.1 years), 78% reported perceiving social isolation. Factors significantly associated with perceived social isolation were female sex (*P* = .001), New York Heart Association class IV (*P* = .001), stress about HF (*P* = .002), paroxysmal nocturnal dyspnea (*P* = .030), edema in the lower limbs (*P* = .001), report of receiving many medications (*P* = .001), change in body image (*P* = .032), and not following limitations in fluid and sodium intake (*P* = .001). The MFIS total score determined moderate to high levels of fatigue (median, 70 points; range, 21–105 points). Total fatigue was statistically significantly associated with social isolation as perceived by patients (*P* = .001). In conclusion, demographic and clinical characteristics as well as fatigue are associated with perceived social isolation. It is essential to evaluate social isolation in routine practice.

## Introduction

Heart failure (HF) is expanding globally at an alarming rate mainly due to the aging population and despite better therapeutic treatment of cardiac diseases.^1^ This clinical syndrome affects approximately 26 million people^1^ worldwide, and >960,000 new cases are being diagnosed each year.^2^ Currently, in the United States, >6 million individuals suffer from HF, and this number is expected to increase by 8 million by 2030.^2^ In Europe, the observed variations in prevalence, incidence, and morbidity rates are mainly attributed to diversities in patients’ characteristics or in the methodology of research studies.^1^ This progressive disease is associated with an increased risk of mortality, elevated health care use,^1,2^ and diminished quality of life.^3^

Social isolation is defined as the absence of social interactions and relationships with family or friends.^4,5^ The influence of subjective and objective social isolation on the risk of mortality is comparable to well-established risk factors.^6,7^

HF patients are vulnerable to social isolation as they tend to be older, have comorbidities, or have few social ties due to life course factors like retirement. Perceived social isolation in HF is associated with an increased risk of emergency department visits,^2^ 90-day rehospitalization,^8^ and elevated health care utilization.^9^

Interestingly, social isolation is associated with vital exhaustion, which is characterized by excessive fatigue, feelings of demoralization, and increased irritability.^10^ Moreover, an increase in fatigue indicates an increase in deterioration of the quality of life ^11^ Clinical outcomes in HF depend also on psychological stress in the form of anxiety and depression,^12^ while the more the support, the less the anxiety.^13^

Evaluating perceived social isolation during follow-up visits might be a step to identifying this vulnerable group of patients and providing holistic management.

At research and clinical levels, the assessment of perceived social isolation is necessary when planning interventions to improve HF outcomes.

The aim of this cross-sectional study was to evaluate the factors associated with perceived social isolation and to assess the impact of fatigue on social isolation.

## Materials and methods

### Study population

In the present cross-sectional study, we recruited 100 HF patients who visited outpatient clinics for routine follow-up by convenience sampling. Criteria for inclusion of patients in the study were as follows: (1) age > 18 years; (2) diagnosis of HF as assessed by the cardiologist and confirmed by medical records; (3) ability to write, read, and understand the Greek language; and (4) ability to read and sign the informed consent form. The exclusion criteria were as follows: (1) patients with mental illness, (2) those visiting outpatient clinics to treat other comorbidities and not HF, (3) patients with cognitive impairment and sight or hearing problems, and (4) hospitalized patients.

### Ethical considerations

Written, informed consent for participation was obtained from all patients after explanation of the purpose and procedure of the study. Participation was on a voluntary basis, and anonymity was preserved. Furthermore, all participants were informed of their rights to refuse or discontinue their participation, according to the ethical standards of the 1983 Declaration of Helsinki.

### Procedure

The process of filling out the questionnaires lasted between 15–30 min and took place in a private room to guarantee privacy. Patients participated in the study after their follow-up.

### Data collection

Data were collected by completion of the “Modified Fatigue Impact Scale” in Greek (MFIS-Greek) and a self-report item of whether they perceived social isolation on a 4-point Likert scale (1 = very much, 2 = enough, 3 = a little, and 4 = not at all). Data for each patient also included demographic, clinical, and self-reported characteristics. Participants were also classified according to New York Heart Association (NYHA) classification, which is widely used in the clinical area for HF patients.^14^

### Measuring fatigue of heart failure patients

The MFIS-Greek was used to evaluate fatigue. The scale consists of 21 questions that assess the fatigue of patients in the last month (4 weeks). Respondents answer every question on a Likert-type scale (scores range from 1–5 points). The scale consists of 2 separate groups of questions concerning (1) physical fatigue and (2) mental fatigue, respectively. The score assigned to the questions is summed separately for questions that assess physical fatigue, for those that assess mental fatigue, and all questions together to an aggregate score (the total fatigue). Higher scores indicate greater fatigue. The scale has an overall Cronbach’s α value of 0.960.^15^

### Statistical analysis

Categorical data are presented using absolute and relative (%) frequencies, whereas continuous data are presented with median and interquartile range values as normality did not hold (checked graphically with histograms and Q–Q plots as well as the Kolmogorov–Smirnov tests). A chi-squared test of independence was performed in order to evaluate the association between social isolation and patients’ characteristics.

The Kruskal–Wallis test was used to test the existence of association between social isolation and fatigue. Moreover, multinomial logistic regression was performed to estimate the effect of patients’ characteristics and fatigue on the probability of social isolation. Results are presented as odds ratios (ORs) and 95% confidence intervals (CIs). The level of statistical significance was set at 0.05. All statistical analyses were performed using SPSS version 20 (IBM Corporation, Armonk, NY, USA).

## Results

### Sample description

Of the 100 patients enrolled, the majority were men (68%), and the mean age of the sample was 68.6 ± 7.1 years.

Concerning clinical characteristics, patients who had NYHA class IV accounted for 44% of the sample. The majority of patients (70%) reported experiencing stress about their HF course and having paroxysmal nocturnal dyspnea (71.7%) and edema in the lower limbs (77.6%).

In terms of self-reported characteristics, 84.8% reported receiving many medicines, 83.8% reported a change in body image, and only 20.2% had limited fluid and sodium intake at the level of very much **(Table 1)**.

**Table 2** presents results related to fatigue of HF patients and whether patients declared they perceived social isolation.

In terms of fatigue, ≥50% of patients scored <70 points (median) in the total score of fatigue and <43 points and 28 points for physical and mental fatigue, respectively. Regarding the total score, it was found that 25% of the participants had a score of >81 points. Accordingly, with regard to physical and mental fatigue, 25% of the enrolled patients had scores of >49 points and >33 points, respectively. These values indicate moderate to high effects of HF on fatigue.

Lastly, patients who perceived social isolation due to HF at the level of very much or enough accounted for 40.0% and 38.0% of the sample, respectively.

### Associations between perceived social isolation and patients’ characteristics

**Table 3** presents the associations between perceived social isolation and patients’ characteristics.

Perceived social isolation in HF patients was statistically significantly associated with sex (*P* = .001), NYHA class (*P* = .001), stress about HF (*P* = .002), paroxysmal nocturnal dyspnea (*P* = .030), edema in lower limbs (*P* = .001), whether they reported receiving many medications (*P* = .001), whether they reported a body change (*P* = .032), and whether patients had limited fluid and sodium intake (*P* = .001). More specifically, patients who perceived social isolation at the level of “very much” were mostly women (55.3%), had NYHA class IV more frequently (57.9%), had stress about HF (78.9%), had paroxysmal nocturnal dyspnea (84.2%), had edema in lower limbs (89.5%), reported receiving many medicines (97.4%), reported experiencing body changes (94.7%), and reported enough limited salt and fluid intake (44.7%).

**Table 4** presents the associations between perceived social isolation and patients’ fatigue. Total fatigue was statistically significantly associated with perceived social isolation as reported by patients (*P* = .001). Patients who perceived social isolation at a level of “very much” had higher scores in total, physical, and mental fatigue (median scores, 47, 29, and 49.5 points, respectively) than patients who perceived social isolation “a little or not at all” (median scores, 36, 18, and 36 points, respectively).

### Impact of fatigue and patients’ characteristics on social isolation

Multinomial logistic regression was performed in order to assess the effect of fatigue and patients’ characteristics on perceived social isolation, and the results are presented in **Table 5**. Female patients had a 24.67 times greater chance than male patients of perceiving social isolation “very much” compared to a little/not at all (OR, 24.67; 95% CI, 1.54–394; *P* = .023).

Patients with edema in their lower limbs had 17 and 16.92 times greater chances than those with no edema in their lower limbs of perceiving social isolation “very much” or “enough,” respectively, compared to a little/not at all (OR, 17.00; 95% CI, 1.19–243.37; *P* = .037 and OR, 16.92; 95% CI, 1.24–231.14; *P* = .034, respectively).

Lastly, an increase of 1 point in mental fatigue corresponded to a 26% increase in the chance of a patient perceiving social isolation “very much” compared to “a little” (OR, 1.26; 95% CI, 1.01–1.58; *P* = .049).

## Discussion

In the present study, the majority of patients reported perceiving social isolation. Several factors might be responsible for this finding, depending on the demographic and clinical characteristics of the participants. As the majority of the sample studied were classified into NYHA class III/IV, it is possible that they experience functional impairment due to the severity of symptoms. The age of the participants (68 ± 7.1 years) might explain other difficulties, such as living alone or the loss of family and a supportive environment. Given that HF is predominately a disease of the elderly, hearing or vision deficits may contribute to their social isolation in addition to fatigue. The descriptive result that 84.8% reported receiving many medicines might imply other comorbidities. A proportion of patients reported not to have limited fluid or sodium intake, which might reflect their low adherence to therapeutic advice. All these parameters explain separately or in combination the levels of perceived social isolation.

Furthermore, patients with HF of NYHA class IV experience a poor quality of life.^16^ Meanwhile, the severity of NYHA class II may be perceived as mild or unalarming, by definition, to treat.^17^ The crucial point is not to cite the prevalence of isolation but to increase awareness about this determinant. Patients who experience high levels of social isolation have a >3.5 times elevated risk of death as well as 1.7 and 1.6 times higher risks of hospitalization and emergency department visits, respectively.^2^ Moreover, social isolation is related to a 55% greater risk of hospital readmission (relative risk, 1.55).^18^ The issue of social isolation among HF is not a recent one. A relevant prior study among 119 clinically stable HF outpatients (mean age, 65.7 ± 9.6 years) revealed social isolation as a predictor of mortality after controlling for depressive symptoms, failure severity, functional status, and age (relative risk, 1.36).^19^

In terms of clinical characteristics, social isolation was associated with NYHA class IV, paroxysmal nocturnal dyspnea, and edema in the lower limbs. A possible explanation is that patients felt unable to maintain a rhythm of social life due to limitations imposed by HF. It is not rare that patients acknowledge the symptom burden as a threat to social integration. Possibly, physical restrictions along with diminished ability to fulfill prior roles may decrease patients’ confidence when planning social integration.^20^ Furthermore, failure of health professionals to spend time with patients or adequately address their needs is a compounder to isolation.^20^ Patients who perceive a decline in functional status report experiencing hopelessness and frustration,^21^ which might deteriorate into social isolation. On the other hand, perceptions of social isolation increase vigilance for threat or heighten feelings of vulnerability to social threats.^2,22^ These threats impair physiological and immunological functioning. As people age, perceived social isolation may increase the risk for depressive symptoms, sleep disturbances, and cognitive decline and overall increase the risk of morbidity and mortality.^2^ Ultimately, patients experience a higher burden of isolation.

Results showed that perceived social isolation was associated with not following fluid and sodium intake recommendations. Possibly, isolated HF patients may be more vulnerable to non-compliance with their therapeutic regimen as they are deprived of support. On the contrary, patients with a high level of support report better self-care, are more likely to consult with a health professional for weight gain, limit amounts of fluids, and take their medication and exercise on a regular basis.^23^

Moreover, body changes were associated with perceived social isolation. Every alteration in physical appearance may adversely affect patients’ adjustment to HF and their motivation for social activities.^24^ Furthermore, participants who report taking a lot of medicines felt isolated. Possibly, this finding may reflect comorbidities or disease severity. However, polypharmacy (use of ≥5 medications) is a well-known issue among HF patients, ranging from 17.2%–99%. Polypharmacy is associated with worse outcomes, including falls, disability, and hospitalization.^25^ This additive burden in combination with social isolation may serve as a reason for discontinuation of medication.

Social isolation was associated with stress about the HF course. Having few social ties or non-frequent social activities may heighten reactions to stress exposure and reduce individuals’ coping abilities, thus having deleterious effects on one’s well-being. Older adults (aged > 50 years) are more likely to experience changes such as retirement and bereavement. Profound losses such as widowhood are associated with increased loneliness, which are a key predictor of mental disorders such as depression.^26^ Social isolation is characterized by feelings of powerlessness, hopelessness, and social dysfunction.^27^

In the present study, isolated participants experienced more fatigue. Results revealed that the 1-point increase in mental fatigue indicated a 26% increase in chances of perceiving social isolation. In the light of this finding, social isolation represents a new area of interest in alleviating fatigue. Anxiety is associated with mental fatigue, whereas depression with reduction of activity, low motivation, and decreased functioning.^28^ Fatigue has a devastating effect on the patient’s ability to cope and manage daily and social activities, including self-care and adherence to recommended treatment. HF patients usually describe the mental aspects of fatigue as demoralizing. On the other hand, fatigue leads to avoiding efforts for social integration and being isolated.^29,30^ Older patients with HF that perceive their disease as debilitating, are gradually compromising their functional status, and often disrupt social functioning.^27^

Fatigue is a circular process in which the consequences of fatigue further exaggerate the experience. However, fatigue could be alleviated by restorative activities.^30^ The assistance provided to socially isolated HF patients varies by the needs of the patient and by available patient and community resources such as psychological or social services or referral to specialized services to meet patient needs.^2^

Social isolation is associated with an increased incidence of cognitive and functional decline, worse health-related quality of life, and increased mortality risk, which are described in both hospitalized and ambulatory HF patients.^27^ Therefore, practical approaches to reduce isolation in HF are essential, which include establishment of constant communication, exhibition of human and honest interest about patients’ lives, and screening and keeping a registry of high-risk patients. Clinical encounters are a crucial time to screen and identify patients who are at risk or experience isolation.^2^ Telemonitoring and telemedicine, which are not hindered by economic, geographic, and bureaucratic barriers, are an alternative option to support and promote care to this vulnerable group of patients.^31^ However, the question regarding the familiarity of modern technology in the elderly population remains. Guideline-directed medical therapy improves clinical outcomes and survival in HF patients, but many elderly patients are excluded from clinical studies due to their age, comorbidities, functional or cognitive impairments, polypharmacy, and the high risk of rehospitalization after hospital discharge.^32^ Last but not least, transition care programs including home visits alone or in combination with telephone calls may enhance the continuity of care and the quality of life among HF patients.^33^

### Limitations of the study

Limitations of this study include the cross-sectional design and the use of self-reporting instruments. Convenience sampling is one of the limitations as this method is not representative of all populations with HF living in Greece, thus limiting the generalizability of the results. The sample size was relatively small, although many significant associations were observed. Moreover, there was no next measurement in time that would allow the evaluation of possible changes in all dimensions under assessment (fatigue, perceived social isolation). Furthermore, it is important to consider other confounders that were not a subject of inquiry in the present study but are shown to have an effect on isolation such as cognitive impairment, depression, and low self-care.

## Conclusions

Results showed that 78% of the participants perceived social isolation, which was associated with sex, NYHA class IV, stress about HF course, paroxysmal nocturnal dyspnea, edema in the lower limbs, receiving many medicines, belief of body changes, and following no limitations in fluid and sodium intake. Fatigue was associated with perceived social isolation.

Female patients had a 24.67 times higher chance than male patients of perceiving social isolation and patients with edema in their lower limbs had 17 and 16.92 times higher chance of perceiving social isolation. Lastly, an increase of 1 point in mental fatigue indicates a 26% increase in the chance of perceiving social isolation.

An in-depth understanding of perceived social isolation is important to design future interventions which would enhance social connectedness.

Instead of putting emphasis on isolation, the solution is to build opportunities for social interaction.

## Figures and Tables

**Table 1:** Patients’ Characteristics (N = 100)

	n (%)
NYHA	
II	20 (20%)
III	36 (36%)
IV	44 (44%)
Do you experience stress about your HF course (yes)	70 (70%)
Do you have paroxysmal nocturnal dyspnea? (yes)	71 (71.7%)
Do you have edema in lower limbs? (yes)	76 (77.6%)
Are you taking many medicines?	84 (84.8%)
Did you experience a change in body image? (yes)	83 (83.8%)
Have you limited fluid and sodium intake?	
Very much	20 (20.2%)
Enough	51 (51.5%)
A little	21 (21.2%)
Not at all	7 (7.1%)

*Abbreviations:* HF, heart failure; NYHA, New York Heart Association.

**Table 2:** Measuring Fatigue of HF Patients and Perceived Social Isolation

	Median (IQR)	Mean ± SD
Fatigue, points		
Total score (range, 21–105)	70 (61–81)	69.1 ± 17.2
Physical (range, 11–55)	43 (37–49)	41.6 ± 9.4
Mental (range, 10–50)	28 (21–33)	27.6 ± 9.9
	**n (%)**	
Do you perceive social isolation?		
Very much	40 (40%)	
Enough	38 (38%)	
A little	12 (12%)	
Not at all	10 (10%)	

*Abbreviations:* HF, heart failure; IQR, interquartile range; SD, standard deviation.

**Table 3:** Factors Associated with Perceived Social Isolation of Patients with HF

	Perceived Social Isolation			
	Very Much n (%)	Enough n (%)	A Little/Not at All* n (%)	*P* Value
Sex				.001**
Male	17 (44.7%)	25 (65.8%)	21 (91.3%)	
Female	21 (55.3%)	13 (34.2%)	2 (8.7%)	
NYHA				.001**
I–III	16 (42.1%)	17 (47.2%)	23 (100.0%)	
IV	22 (57.9%)	19 (52.8%)	0 (0.0%)	
Stress about HF				.002**
No	8 (21.1%)	9 (23.7%)	14 (60.9%)	
Yes	30 (78.9%)	29 (76.3%)	9 (39.1%)	
Do you have paroxysmal nocturnal dyspnea?				.030**
No	6 (15.8%)	11 (28.9%)	11 (47.8%)	
Yes	32 (84.2%)	27 (71.1%)	12 (52.2%)	
Do you have edema in your lower limbs?				.001**
No	4 (10.5%)	6 (15.8%)	12 (54.5%)	
Yes	34 (89.5%)	32 (84.2%)	10 (45.5%)	
Are you taking many medicines?				.001**
No	1 (2.6%)	3 (7.9%)	11 (47.8%)	
Yes	37 (97.4%)	35 (92.1%)	12 (52.2%)	
Did you experience a change in body image?				.032**
No	2 (5.3%)	7 (18.4%)	7 (30.4%)	
Yes	36 (94.7%)	31 (81.6%)	16 (69.6%)	
Have you limited fluid and sodium intake?				.001**
Very much	2 (5.3%)	7 (18.4%)	11 (47.8%)	
Enough	19 (50.0%)	21 (55.3%)	11 (47.8%)	
A little/not at all	17 (44.7%)	10 (26.3%)	1 (4.3%)	

*Abbreviations:* HF, heart failure; NYHA, New York Heart Association. *Statistically different category compared to “very much,” after correction for multiple comparisons. **Statistically significant.

**Table 4:** Associations Between Perceived Social Isolation and Patients’ Fatigue

	Perceived Social Isolation			
	Very Much Median (IQR)	Enough Median (IQR)	A Little/Not at All Median (IQR)	*P* Value
Fatigue, points				
Total	47 (41–50)*	45 (37–49)	36 (19–41)	.001**
Physical	29 (25–35)*	28.5 (22–33)	18 (13–31)	.002**
Mental	49.5 (44–57)*	49 (43–53)	36 (32–40)	.001**

*Abbreviation:* IQR, interquartile range. *Statistically different score than patients with a little/not at all limitation of social contacts, after correction for multiple comparisons. **Statistically significant.

**Table 5:** Impact of Fatigue and Patients’ Characteristics on Perceived Social Isolation

	Perceived Social Isolation Reference Category: A Little/Not at All			
	Category: Very Much		Category: Enough	
	OR (95% CI)	*P* Value	OR (95% CI)	*P* Value
Sex				
Male	Ref. Cat.		Ref. Cat.	
Female	24.67 (1.54–394.40)	.023**	6.91 (0.44–107.50)	.168
NYHA				
I–III	Ref. Cat.		Ref. Cat.	
IV	*	.992	*	.990
Stress about HF course				
No	Ref. Cat.		Ref. Cat.	
Yes	6.21 (0.50–76.34)	.154	5.70 (0.52–62.61)	.164
Paroxysmal nocturnal dyspnea				
No	Ref. Cat.		Ref. Cat.	
Yes	0.43 (0.02–7.64)	.565	0.16 (0.01–2.49)	.190
Edema in lower limbs				
No	Ref. Cat.		Ref. Cat.	
Yes	17.00 (1.19–243.37)	.037**	16.92 (1.24–231.14)	.034**
Are you taking many medicines?				
No	Ref. Cat.		Ref. Cat.	
Yes	29.37 (0.35–2,497.39)	.136	15.25 (0.36–646.39)	.154
Change in body image				
No	Ref. Cat.		Ref. Cat.	
Yes	0.11 (0.00–3.85)	.226	0.05 (0.00–1.18)	.064
Limit fluid and sodium intake				
Very much	Ref. Cat.		Ref. Cat.	
Enough	4.86 (0.31–77.07)	.262	1.89 (0.16–22.63)	.615
A little/not at all	12.46 (0.40–387.39)	.150	2.71 (0.10–70.33)	.549
Fatigue				
Physical	1.00 (0.83–1.20)	.986	0.94 (0.78–1.12)	.483
Mental	1.26 (1.01–1.58)	.049**	1.24 (0.99–1.55)	.065

*Abbreviations:* CI, confidence interval; NYHA, New York Heart Association; OR, odds ratio; Ref. Cat, reference category. Goodness of fit: Cox–Snell 0.571 indicating good fit of the model, likelihood ratio test for the final model, *P* < .001. *Omitted due to no sample size. **Statistically significant.
